# Supplementary material for: Comparative genomics provides new insights into the diversity, physiology, and sexuality of the only industrially exploited tremellomycete: Phaffia rhodozyma
Source: BMC Genomics. 2016 Nov 9;17:901. doi: 10.1186/s12864-016-3244-7 (PMC5103461; doi:10.1186/s12864-016-3244-7)
Supplement: Additional file 6: — List of orphan genes with links to PFAM (related to Additional file 1: Table S1). (ZIP 1428 kb) [file 12864_2016_3244_MOESM6_ESM.zip › PFAM_UNIQUE/G02259_P.html]

#                                                                            --- full sequence --- -------------- this domain -------------   hmm coord   ali coord   env coord
  
# target name        accession   tlen query name           accession   qlen   E-value  score  bias   #  of  c-Evalue  i-Evalue  score  bias  from    to  from    to  from    to  acc description of target
  
#------------------- ---------- ----- -------------------- ---------- ----- --------- ------ ----- --- --- --------- --------- ------ ----- ----- ----- ----- ----- ----- ----- ---- ---------------------
  
G02259\_P             -            136 Chon\_Sulph\_att       PF06566.6    253     0.013   13.8   1.4   1   1   2.6e-06     0.016   13.6   1.4   104   207     3   102     1   127 0.61 - Chondroitin sulphate attachment domain  
G02259\_P             -            136 NMDAR2\_C             PF10565.4    681    0.0092   13.4   0.0   1   1   6.2e-06    0.0092   13.4   0.0   404   448    49    99    22   133 0.73 - N-methyl D-aspartate receptor 2B3 C-terminus
